# WHO and artificial intelligence: contesting global health futures through foresight

**DOI:** 10.3389/fpubh.2025.1659980

**Published:** 2025-11-12

**Authors:** Jason Tucker

**Affiliations:** 1 Institutet for Framtidsstudier, Stockholm, Sweden; 2 AI Policy Lab, Department of Computing Science, Umeå University, Umeå, Sweden

**Keywords:** WHO, artificial intelligence, AI, narratives, global health, policy discourse, foresight, futures

## Abstract

The article examines the World Health Organization’s (WHO) discourse on artificial intelligence (AI) in their foresight exercises, doing so by drawing on the analytical framework of strong and weak AI narratives. The analysis finds that strong AI narratives (those which depict AI as human-like or even super-intelligent, emphasising existential risks and transformative power) are rarely found. In contrast, the exercises produce a broad range of weak AI narratives (those that emphasise the technical limitations, ethical concerns, and practical governance of specific AI applications in healthcare). The findings reveal how certain AI technologies are foregrounded by WHO, how these are framed as in isolation from other emerging technologies, how this isolation is strategically blurred, and the role of expert participation in legitimising WHO’s policy on AI. Situated within WHO’s broader policy discourse on AI, the paper draws out how the foresight exercises strategically construct and validate particular trajectories aligned with WHO’s existing priorities. Through selective narrative framing, expert input, and methodological design, WHO reinforces its epistemic authority by guiding the discourse on AI in global health toward context-sensitive and manageable use cases of the technology. Ultimately, these foresight exercises serve as a site of contestation, where competing visions of AI in global health are negotiated, and WHO’s influence over future governance in the area is actively shaped.

## 1 Introduction

The hype around artificial intelligence (AI) in healthcare poses a considerable challenge for those working to ensure that this technology aligns with our current and future global health needs. With dominant techno-solutionist discourses claiming that AI is a magic pill for all healthcare needs, there has been an acceptance and normalisation that the increasing scale, scope and speed of the adoption of AI in healthcare is not only a unique means to tackle our healthcare needs, but also inevitable (1, 2). This promise has fuelled a considerable disruption in the global political economy of health, one that sees a recasting of power relations between old and new actors in the field.

The World Health Organization (WHO), as an epistemic authority on global health, serves as an agenda-setter for the use of emerging technology in the healthcare sector (3). As such, WHO is at the frontline of trying to manage the disruptive nature of AI in global health, while simultaneously seeking to facilitate its development and adoption where it is, or could be, beneficial (4). While previously WHO has taken a more reserved position about the future role of AI in global health, this changed during the COVID-19 pandemic, when the organization pivoted to being significantly more positive about its potential to revolutionise global health (5). This is a position they continue to hold today. In this regard, WHO have been active in trying to shape the global governance of AI and reassert their role as critical mediators between the technology and society. For example, WHO have provided specific actionable guidance to healthcare actors on how to regulate or adopt AI, as well as being a trend setter in producing ethical guidelines on the global governance of AI (6).

This is in part a reaction to WHO’s need to shape the discourse and trajectory of AI in global healthcare to ensure it aligns with the organization’s normative and operational goals. However, doing so is notoriously complex given the hype around AI which obscures probable future applications, and how advances in AI would interact with other emerging technologies and trends with which WHO must contend.

One means by which WHO have tried to meet this challenge is through a series of foresight exercises. Foresight provides a framework for the exploration of future scenarios, and thus strategic areas for research and development, in a systematic manner (39). As WHO note: “The aim of the WHO global health foresight function is to inform decisions about innovation, development and emerging science and technology, shape development, diffusion and adoption of health innovations and proactively identify emerging problems and challenges” (World Health Organization, 2023, p. 19).

This matters as the foresight exercises reproduce or recast existing WHO policy positions. Doing so does not merely have normative ramifications, but has real world implications. WHO can use the outcomes of these exercises to justify their recommended use or rejection of, often poorly governed, AI technologies in global health. WHO’s approval has a significant impact on which technologies will be sought, and approved, by local, national, and international actors in healthcare. The foresight documents are thus meaning-making as well as being what Webb et al. (7) referred to as a physical trace of a social setting.

To explore how foresight is used by WHO to frame AI, a discourse analysis of all eight WHO foresight documents published between 2021 and 2024 was undertaken. The research drew on Bory et al. (8) analytical framework of “strong” and “weak” AI narratives. Strong AI narratives depict AI as human-like or even super-intelligent, emphasising existential risks and AI’s transformative power, thus requiring our urgent attention. Weak narratives focus on specific, everyday applications of AI contextualised within broader socio-technical systems (8). The analytical framework of strong and weak AI narratives was used to explore the discourses within the foresight exercises and how this relates to WHO’s role in shaping the current and future role of AI in global health more broadly.

The findings reveal that strong AI narratives in the documents are rare and confined to introductory framing. They serve only to justify the urgency of the exercises themselves. In contrast, the substantive content of these documents prioritises weak AI narratives, which describe specific, narrow applications of AI within health systems. These weak narratives identify a more grounded, critical perspective, highlighting implementation challenges such as data quality, infrastructure limitations, bias, and equity. Interestingly, AI itself is often siloed from other emerging technologies, being framed as having limited scope for application and impact. However, at other times AI is framed as a key enabler for the realisation of a range of other innovations. Further, to this, the role of experts in the foresight exercises in terms of materialising, but also, and critically, legitimising, various AI narratives was identified.

By situating these findings within the broader context of WHO’s policy discourse on AI, the purpose of these foresight exercises, regarding of what they meant to do in the world, is explored. Through the selective framing of specific AI technologies and by controlling the foresight process, it is argued that WHO can be seen as using the exercises to reinforce their epistemic authority in global health. They are doing so by seeking to frame the findings to align with and support their ongoing discourse on AI, and using weak narratives as a means to do so. Thus, the foresight exercises are a site of tension, where the organization can challenge the dominant techno-solutionist discourses on the future of AI in global health, and reassert its legitimacy.

The paper is organised into four sections. It begins by reviewing the role of foresight in global health policy and situating it within WHO’s institutional engagement with futures thinking. Following this the analytical framework of strong and weak AI narratives and the methodology are set out. The main findings of the analysis are then presented, focusing on four key areas. The discussion section then reflects on the implications of these findings in relation to WHO’s broader policy discourse on AI, as well as the role of foresight exercises themselves.

## 2 Background

### 2.1 Foresight, public policy and global health

“Foresight is a systematic process of developing strategic intelligence, mobilising understandings of the past and structured articulation of potential futures – to aid decision making” (9, p. 1).

Through the rigorous imagining of futures, foresight exercises allow participants to confront their collective and individually held norms, assumptions and biases, and in so doing better understand their present-day situation and possible futures (10–13). Whereas futures approaches can envision a range of possible futures, foresight is more concerned with the probable and plausible scenarios. This makes it of use to decision makers given that it provides the ability to shape and not only react to events (14). Regarding emerging technology, this forward-looking process allows one to identify areas for strategic research to achieve desirable technologies (11).

While there are challenges in linking foresight findings to informing policymaking (9), the ability to elevate certain future issues to an institutional level can be highly beneficial for organisations (15). For example, it can be a tool to reduce barriers, such as lack of support from senior decision makers, thematic silos and conflicts of interest, as well as barriers related to individual agency (i.e., cognitive biases) (16). Regarding health policy, research has shown that there is currently limited use of foresight approaches in decision making, despite its potential to increase democratic participation, broaden engagement and challenge power imbalances (17).

Policy debates often exhibit what has been described as “presentism,” with time-constrained policy development becoming the norm (15). This tendency is even more pronounced in the context policymaking related to AI. While delays in policy formulation are not uncommon, the rapid advancements in AI (both real and perceived), particularly in global health, presents complex challenges for those wishing to guide its development. In this context, foresight is proposed as a useful approach to support policymakers. This is because foresight can be understood as the negotiated presentation of futures, and as such serves an agenda setting function. Being the epistemic authority on global health, WHO’s engagement with foresight provides a window, and a rich empirical site, to explore how WHO seeks to shape discourses around the current and future use of AI in global health.

### 2.2 WHO and foresight

In 2020 WHO’s Science Division established a global health foresight function, operated from the Research for Health Department (18, p. 3). The ostensible goal of WHO’s foresight exercises on emerging technologies is to “help Member States better anticipate and prepare for a changing world, to accelerate and fully harness the gains from emerging technologies, while monitoring the risks and challenges that might arise from those technologies” (19, p. 2). This includes the promotion of “appropriate” health interventions using cutting edge technology, efforts toward anticipatory governance and identifying “sustainable financing models” for these technologies (20, p. 23, 46, 55).

Importantly, these foresight exercises extend beyond national governments. WHO Science Division’s aims to increase the use of these approaches for a range of actors and at a range of scales, including at the WHO organisational level, to build “futures thinking” into strategic health planning (18, p. 3). Futures thinking is thus posited as a vital means by which WHO staff, and the organisation, can successfully anticipate and response to rapid changes, as well as to critically reflect on their current operations (18, p. 5).

WHO’s focus on anticipatory governance is nothing new. It has been a feature of their organisational toolkit for over a decade. As WHO described in 2012 “Anticipatory governance with participatory foresight mechanisms can also increase social resilience by shifting policy focus from risks to addressing more fundamental systemic challenges and deliberating the social aspects (such as values) of public policy and science (such as evidence) jointly” (21, p. xii). This approach aligns with the broader field connecting anticipatory governance and futures thinking (22, 23).

The inclusion of AI as one area of focus of foresight exercises by WHO is a rather new development, and one that warrants attention. WHO’s position *vis a vis* AI can be seen to have shifted, from framing AI as a risk to be managed, to one where it is seen as a potential saviour for a plethora of global health challenges (5). Exploring this shift is important for several reasons. First, WHO is an epistemic authority on global health. Their discourse on AI serves a normative function, reflecting how they aim to manage the risks and reap the benefits of AI in global health, and their role in this. However, their discourse has impacts beyond the organisation. For example, when looking at the discourses on AI in healthcare Shipton and Vitale (24) claim that it is not only what is said but also what is omitted, with research on the “politics of avoidance” highlighting how AI narratives can obscure upstream causes of health challenges and health inequalities. WHO’s discourse on AI thus can have an impact on how healthcare is understood and addressed more broadly: Though as Regis et al. (25) note, measuring the normative impact of WHO is challenging. Additionally, previous research has shown how discourses on AI shape emerging governance structures (26). With global governance structures around AI in health still in a developmental phase, exploring WHO’s discourse on AI is therefore timely and important.

Second, the discourse on AI in these foresight exercises does not only serve a normative function. It also reflects, reinforces, and recasts existing WHO policy positions on AI. Given the authority that WHO holds, these discourses have real-world consequences. As such, these foresight documents should be considered part of a wider discourse where WHO legitimise their support for, or opposition to, the use of various AI technologies in various contexts. Further to this, local, national and international actors look to WHO for guidance in this area. AI technologies seen as being approved of by WHO can be funded, developed and deployed in areas where there is still only emerging or limited regulatory oversight. For example, in 2021 WHO released the toolkit *Determining the Local Calibration of Computer-Assisted Detection (CAD) Thresholds and Other Parameters. A Toolkit to Support the Effective use of CAD for TB Screening* (27). In so doing they provided legitimacy for the expanded use of AI assisted CAD in tuberculosis screening, despite a lack of national or global governance on AI to appropriately manage the risks. Indeed, it was only in 2021 that WHO released its broad principles on the *Ethics and Governance of Artificial Intelligence for Health: WHO Guidance* (20).

### 2.3 Strong and weak AI narratives

The analysis presented here draws on the concept of “strong” and “weak” AI narratives set out by Bory et al. (8). This analytical framework was proposed to draw more attention to, and develop sensitivity around, the critical role of the interconnected narratives that shape AI futures. It is argued that such an approach is needed given that “When looking at policy debates and policy documents on AI, there is a remarkable ambivalence between the prevalence of strong and weak AI narratives” (8, p. 7). This approach builds on work on AI imaginaries (for example 28, 29) and proposes a framework to complement existing categorisation of technological imaginaries while addressing the need for more critical interrogation of strong and weak AI narratives.

Strong narratives range from depictions of AI as having human-like faculties, to the more extreme narratives about singularity, existential risk, super-intelligence and AI attaining consciousness. This also includes narratives on AI as a powerful transformative force, requiring urgent action to mitigate the harms that it can cause (8). The academic literature and public debate largely reproduce and engage with strong narratives. This has been the subject of sustained critique for being overly speculative, obscuring other political motivations and distracting from the current harms caused by AI systems (30, 31).

By contrast, weak narratives are those which detail specific AI applications in specific contexts, situating these as part of a broader socio-technical system (8). Weak narratives focus on and prioritise the everyday, contextual and pragmatic applications of AI. It is the “fictional and non-fictional representations of AI technologies that operate in narrow documents, such as voice assistants, automatic translators, or chatbots” (8, p. 4). This approach acknowledges that these technologies can outperform humans, but critically, only in narrow (weak) applications, thus differentiating them from the strong narrative focus on broad transformative power, such as super/general artificial intelligence. Weakness, as it is used here, should not be conflated with lack of importance or impact. Rather, as the framework sets out, it is a critical site for the investigation of AI given the pragmatic and symbolic ramifications of the technology at this scale (8). This builds upon previous efforts to draw attention to the importance of the everyday aspect of automation (32) and digital health (33), to name but a few examples of a rich body of research.

While this approach seeks to nuance the analysis of AI narratives by paying more attention to the under researched weak narratives, it does not reject the importance of strong narratives. Rather it calls for attention to be paid to the relationship between the two, particularly between their public/private and fictional/non-fictional discourses (8). As such, it points to a critical, though under researched site of contestation and power relations over the role of AI in society.

## 3 Methodology

By focusing on foresight documents, we can move beyond the abstract uncontroversial notions, or what has been referred to broadly as the “thingness” of AI (34). This is because WHO foresight exercises not only seek to better position participants to anticipate the future role of AI in global health, but also to shape it by reinforcing selected narratives. This arises as the exercises demand that the participants be specific about emerging trends and technology, and then assess their likeliness of occurring, chance of adoption, potential impacts and timeframe, doing so as a collaborative exercise in comparison with other emerging trends and technologies.

Regarding the usefulness of documents to explore discourses more generally, they provide a unique, curated, “official” representation of WHO’s policy position on the future of AI in global health. Documents should be considered as “artefacts that are created for a particular purpose, crafted according to social convention to serve a function of sorts. It is this social production (and indeed consumption) of documents that gives them analytical affordance” (35, p, 369). As such, document analysis is a well-established approach in health policy studies (36).

**Figure 1:**
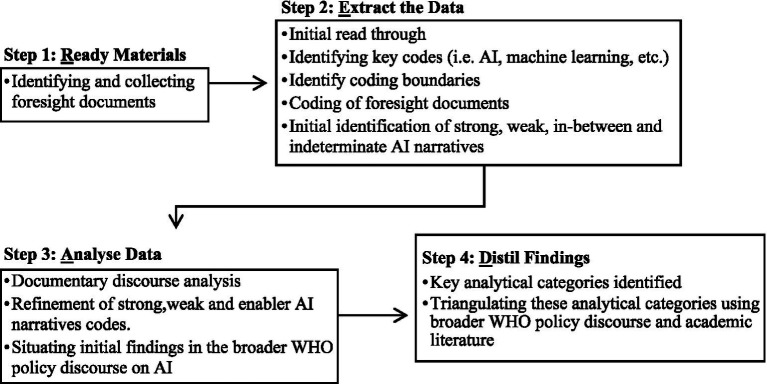
READ approach workflow.

The point of departure was to draw on the analytical framework of strong and weak AI narratives (8), to explore WHO’s discourses on AI within and beyond their foresight exercises. The data was analysed using a multi-stage process call the READ Approach, developed by Dalglish et al. (36) specifically for documentary analysis of health policy. The four steps of this approach (Readying Material, Extract the Data, Analyse Data, and Distil Findings), are used to structure this methodology section (see Figure 1. for a workflow).

### 3.1 Readying materials

The focus of the analysis was on the publicly available official reports produced to guide or document WHO’s horizon scanning/foresight exercises on emerging technologies and future trends. Eight reports published between 2021 and 2024 were analysed. These covered a range of specific and general topics (see Table 1). All reports published as part of WHO’s foresight exercises by the Research for Health Department, found on the WHO publications website before March 2024, were included. WHO’s foresight exercises were a multistage process involving WHO and experts (the role of experts being covered at length in sections *4. Findings* and *5. Discussion*). This process is set out in Figure 1, and more details on the various foresight strategies employed by WHO can be found in *Foresight Approaches in Global Public Health: A Practical Guide for WHO Staff* (18).

**Table 1:** Documents analysed.

Document	Year	References
Emerging Technologies and Dual-use Concerns: A Horizon Scan for Global Public Health	2021	(19)
WHO Consultative Meeting on Science and Technology Foresight Function for Global Health	2021	(43)
Bending the Trends to Promote Health and Well-Being: A Strategic Foresight on the Future of Health Promotion	2022	(44)
Emerging Trends and Technologies: A Horizon Scan for Global Public Health	2022	(39)
Foresight Approaches in Global Public Health: A Practical Guide for WHO Staff	2022	(18)
Imagining the Future of Pandemics and Epidemics: A 2022 Perspective	2022	(42)
Emerging Technologies and Scientific Innovations: A Global Public Health Perspective	2023	(40)
Imagining Futures of 3D Bioprinting	2024	(41)

### 3.2 Extract the data

Initially all the documents were read in chronological order in a process of becoming familiar with the data. The next stage was to identify references to AI, which were then classified as either strong, weak, in-between or indeterminate. Following this, initial codes were identified within these broader categories, which led to the generating of initial themes. These themes were then reviewed, defined and named. Throughout the process the initial classification was reflected upon leaving three main categories, weak, strong, and in-between, with the later of these eventually being reclassified as the enabler function of AI discussed in section *4.3 AI as innovation or enabler*.

With regards to coding boundaries, the identification of “AI” included references to AI/Artificial Intelligence, machine learning, deep learning, natural language processing (NLP) and computer vision. Robotics were included when directly related to AI. There are no references to expert systems, or other fields of AI research, which would have otherwise been included. AI was implicitly involved in other areas of innovation in the documents (i.e., some applications of the Internet of Things). However, it was the explicit references to AI that were the focus of this paper, as it was how these narratives related to the broader WHO discourse of AI and global health that was of interest. This is not to claim that AI is understood as one singular or graspable technology. However, despite its breadth, the concept of AI is a useful umbrella term which provides an ordering function for policy (37).

A further coding boundary was how either weak or strong narratives were identified. This followed the analytical framework proposed by Bory et al. (8). Weak narratives are those where a specific application (real or imagined) and its context or area of application are discussed. Here a critical element is the narrowing of the discussion to focus on the everyday, the mundane and the contextual application of the technology, even if the application itself remains broad. Strong narratives were those where broad claims related to AI achieving human-like faculties, i.e., the concept of singularity, super-intelligence and AI attaining consciousness, or where AI is “positioned as a powerful and external force that policy and policymakers need to address and tame” (8, p. 7). These two categories allow for the assessment of competing claims about AI, and how both strong and weak narratives “mutually contribute to the construction of AI imaginaries” (8, p. 1). Given this connection, at times distinguishing between the two was problematic, leading to the introduction of the in-between and indeterminate categories in the first round of coding. Examples of both weak and strong narrative instances are shown in Table 2.^1^ Instances were a distinct excerpt within the document that articulates either a strong or weak narrative on AI, or where AI was discussed but could not easily be classified as strong or weak (with in-between and indeterminate being used initially, followed by the enabler code later on).

**Table 2:** Examples of instances of strong and weak AI narratives.

Strong AI narrative	Weak AI narrative
“…artificial intelligence, and big data, all of which pose transformational opportunities but also risks ….” (43, p. 5).	“…AI prediction of the 3D structures of proteins” (40, p. 18).
“Global Trend: 4th Industrial revolution incl. AI and robotics.” (44, p. 10).	“Machine learning for antibiotic discovery…” (39, p. 5).

Coding included the instance of the AI narrative, as well as the sentence or paragraph in which it was found, where this was essential for its contextualisation. In some cases, several weak AI narratives instances were identified in one sentence. When this occurs they are counted as sperate instances but analysed in relation to each other. Instances of AI as an enabler was counted separately (see Table 3). The coding was undertaken alone by the author, without using any software. The limitations of solo coding were acknowledged, with triangulation, seeking disconfirming evidence and peer debriefing being undertaken to ensure reliability and to identify and address bias.

**Table 3:** Number of instances of weak and strong AI narratives and AI as an enabler per document.

Document	No. weak AI instances	No. strong AI instances	No. AI as enabler instances
Emerging Technologies and Dual-use Concerns: A Horizon Scan for Global Public Health	1	–	–
WHO Consultative Meeting on Science and Technology Foresight Function for Global	1	1	1
Bending the Trends to Promote Health and Well-Being: A Strategic Foresight on the Future of Health Promotion	–	1	–
Emerging Trends and Technologies: A Horizon Scan for Global Public Health	2	–	1
Foresight Approaches in Global Public Health: A Practical Guide for WHO Staff	–	1	1
Imagining the Future of Pandemics and Epidemics: A 2022 Perspective	11	–	–
Emerging Technologies and Scientific Innovations: A Global Public Health Perspective	19	–	11
Imagining Futures of 3D Bioprinting	–	–	–

In this research WHO is referred to in the singular, which can be seen as potentially flattening a multifaceted global organisation. The research focuses on documents produced by the Research for Health Department. While it is not claimed that this Department represents the whole organisation, given their role as the appointed agenda setter and authority in futures work at WHO, they provide an interesting entry point for understanding broader organisational narrative formation. The findings were also situated within a broader set of policy documents on AI produced by WHO, thus introducing a breadth of contextualisation to avoid this organisational.

### 3.3 Analyse data

The data was analysed to identify and explore, not only the content of the documents regarding weak or strong AI narratives, but also their discursive purpose in framing the current and future role of AI in global health and WHO’s role in this. In line with this the relational priorities of the documents were considered as meaning making in a broader context (35). As such, the documents were seen as “… active agents in the world… and… as a key component of dynamic networks rather than as a set of static and immutable ‘things’” (38, p. 821).

These foresight documents formed the backbone of the research, as the focus was on their use by WHO and the role of weak and strong AI narratives within these. Therefore, the initial analysis began with these documents. However, given the desire to situate the findings within the broader organisational discourse on the future of AI in global health, other WHO documents, including content on their websites and official policy documents were used to contextualise and triangulate the findings. By returning to the data with insights drawn from other WHO publications and the broader literature, I was able to reflect on how these narratives constructed, maintained or contested WHO, and other dominant actors, discourses on the future of AI in global health.

### 3.4 Distil findings

Finally, after the analysis phase, key analytical categories from the data were identified. These, and the findings related to them, are presented in the following section as subheadings. The findings section focuses on the content and intertextuality of AI narratives in the documents and is thus quite descriptive. The discussion section reflects on how the strong and weak narratives in and between these foresight exercises can be understood within the broader discourse of AI and global health that WHO is producing, reproducing or challenging.

As Dalglish et al. (36) note, this final stage of the research should ideally result from reaching data saturation and completeness regarding the data extraction and analysis. This was the case here, where the materials on the foresight exercises were limited. At this stage, and throughout, the validity of the findings was supported through triangulation using other WHO documents and reliable sources. Quotes from the data were selected as representative of broader points of interest.

### 3.5 Limitations

As discussed in section *3.2 Extract the Data* limitations existed regarding the potential for bias and reliability in solo coding. A further limiting factor was that the weak AI narratives that make it to the final dissemination documents can introduce survivor bias. This is true in a sense and, while not publicly available, a review of the selection process, including those AI narratives that were removed would be a valuable addition to the findings of this research. However, given the focus here on how certain AI narratives are presented and justified in these documents, it is an exploration of the survivors that is of interest. Nevertheless, it is recognised that this has its limitations. If made available, follow up work on the negotiations behind these documents would be highly enlightening regarding the formation, contestation and use of strong and weak AI narratives in WHO discourse on AI.

## 4 Findings

### 4.1 Abundant weak and rare strong AI narratives

Regarding the instances of weak AI narratives, these were present in five out of eight of the documents (19, 39–41, 43) with the most being found in the foresight exercises with a broader focus, i.e., those on emerging technologies (see Table 3). Weak AI narrative instances were concentrated in two documents, with 19 instances in *Scientific Innovations: A Global Public Health Perspective* (40) and 11 in *Imagining the Future of Pandemics and Epidemics: A 2022 Perspective* (42). Two instances of weak AI narratives were found in *Emerging Trends and Technologies: A Horizon Scan for Global Public Health* (18), and one in both *Emerging Trends and Technologies: A Horizon Scan for Global Public Health*. These weak narratives pointed to AI being most potentially impactful in (1) diagnostics (including precision medicine), (2) drug discovery, (3) clinical reasoning support (4) health system management (including public health), (5) digital twins, (6) disease outbreak management and (7) artificial organs. These all varied considerably in terms of likelihood of being realised and potential impact, though the time frame was 5–10 years for the vast majority (other timeframes being 0–5 and 10 + years). Instances on strong AI narratives only occurred three times, once in each of the follwing, *WHO Consultative Meeting on Science and Technology Foresight Function for Global Health* (43), *Bending the Trends to Promote Health and Well-Being: A Strategic Foresight on the Future of Health Promotion* (44) and *Foresight Approaches in Global Public Health: A Practical Guide for WHO Staff* (18).

**Figure 2:**
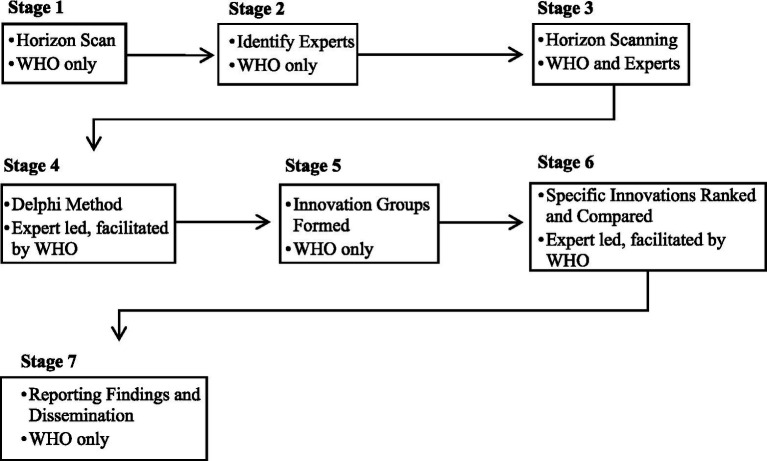
The stages of the foresight exercises (including if this was led by WHO, the experts or a collaboration).

Given the nature of the foresight exercises, as set out in Figure 2, AI applications which were seen as unlikely to occur or would have limited impact were excluded. This exclusion was formalised in the process, being undertaken at stages 3, 5 and 6 (how this related to the role of the experts will be discussed in section 4.4). Only those weak AI narratives deemed to reach a certain threshold of likelihood, chance of adoption or potential impact compared to other emerging trends and technologies were brought forward for more rigorous critical reflection in the foresight exercises. As such, critical reflection related to the application of this technology was built into the process. For this reason, when claims regarding the potential of various AI innovations were made, this was nearly always followed by reflections on its limitations. For example, when discussing AI assisted clinical support systems it was noted that:

“Numerous studies and systematic reviews have been conducted on clinical reasoning support systems. Recent reviews concluded that the impact on clinical care is small to moderate, at least so far. Issues raised about the use of these systems include the reproducibility, reliability and quality of data, especially in countries without the appropriate infrastructure. Other concerns are algorithmic biases, workflow disruption, over-reliance and, conversely, ‘alert fatigue’, the cost of introduction and operation and integration into the training of health professionals” (39, p. 7).

In general, the main limiting factors of AI technologies development and impact were lack of good quality data, the danger of bias, the lack of resources, friction in implementation given legacy systems and challenges in ensuring equitable roll out of the technology.

As mentioned previously, strong AI narratives were rare in the documents. Only three instances were identified in three sperate documents (18, 43, 44), with two of these being the identical text (18, 43). These instances were found in the preamble or introduction of the documents. They were used to frame the need for WHO to act on AI, and to justify the foresight exercises. For example, the “WHO’s normative guidance will be informed by developments at the frontier of new scientific disciplines such as genomics, epigenetics, gene editing, artificial intelligence, and big data, all of which pose transformational opportunities but also risks to global health” (18, p. 5). The possibility of super intelligence or general-purpose AI was not raised in the documents. The role of AI can largely be summarised as playing a supportive role or “augmenting human capabilities” (42, p. 43).

### 4.2 The siloing of AI

When engaging in the materialisation of weak AI narratives, WHO grouped AI together in an Innovation Group with the Internet of Things, wearables, tele-health, augmented and virtual reality^2^ (40). The demarcation of these Innovation Groups was an explicit culmination of all the proceeding reports, except for *Imagining the Future of Pandemics and Epidemics: A 2022 Perspective* (40). Further to this, there was limited overlap between AI and the other areas of innovation (both technical and non-technical) in the foresight exercise documents. AI was largely siloed off from other areas. Instances of where AI narratives were raised in the other Innovation Groups included, managing water storage systems, machine learning for digital health tools, as an enabler of other innovations (discussed in the next section), robotics for training healthcare staff, and machine learning for public health surveillance.

Further to this, beyond the umbrella term of AI, only machine learning, deep learning, NLP (only mentioned once unlike the others), computer vision and robotics (where directly related to AI) were specified. The lack of inclusion of other common AI technologies in healthcare, such as expert systems, is noteworthy and will be elaborated upon further in section 5.2 *Siloing AI and hedging their bets*.

### 4.3 AI as innovation or enabler

The relationship between weak and strong AI narratives is also crucial to understanding how these narratives function and the purpose they serve (8). The data revealed an interesting co-dependence, where AI is seen as both a specific technology that will be transformative for certain areas of healthcare, as well as its development being vital to unlock other broadly defined transformations in the sector. Fourteen instances of AI as an enabler were found in four of the documents (18, 39, 40, 43). Eleven of these were in *Emerging Technologies and Scientific Innovations: A Global Public Health Perspective* (40) (also being the document with the largest number of instances of weak AI narratives). One instance of AI as an enabler was also found in each other following, WHO Consultative Meeting on Science and Technology Foresight Function for Global Health (43), Emerging Trends and Technologies: A Horizon Scan for Global Public Health (39) and Foresight Approaches in Global Public Health: A Practical Guide for WHO Staff (18).

The documents differentiate between “innovation” and “enabler” categories to do this. AI falls into both categories, often with an unresolved “chicken or egg” dependency issue. For example, Big Data analytics is used as a case where AI could be used to significantly improve workflows (40). Yet, it is also noted that the likelihood of this being realised is greatly hampered by legacy systems that perpetuate inefficient workflows and limit the use of electronic health records (part of Big Data) which in turn reduces the impact of current AI interventions (40).

The AI technologies found in the innovation category can all be identified as weak AI narratives. AI as an enabler, however, finds the narratives occupying an in between space, being both weak and strong. Yet, even as an enabler, critical reflection on the implementation of the transformation, more in line with the weak AI narratives approach, was common. For example, “The move toward the use of machine learning and digital services as enabling tools is accompanied by similar challenges and problems, which include biases in data, the reproducibility of data, the problem of explaining ‘black box’ algorithmic decisions, data privacy and potential vulnerability to cyberattacks” (39, p. 12). Further to this, AI was not top of the list for “Relevant” technological innovations. In one exercise 79 percent noted that AI (and machine learning) were of high or very high relevance as a technological innovator, below *Big Data* (88 percent) and *Cloud Computing* (84 percent) and, interestingly, the same percentage as *Building and Sustaining Broad Health Infrastructure* (40, p. 41).

### 4.4 The role of experts

The role of the experts in the foresight exercises was considered as it related to authorship and authority. Authorship matters, and in the documents WHO is listed as the “anonymous” author. However, there are “indications of authorship” i.e., lists of the names of experts involved at various stages and their affiliations, which is used to reinforce claims of authority and legitimacy (35, p.375). The experts were described as those holding expertise in various areas of global health and technology^3^ representing a diversity of age, gender, geographic location etc. Twenty to thirty experts were present in *Stage 3 Horizon Scanning*, *Stage 4 Delphi Method Study*, and *Stage 6 Specific Innovations Ranked and Compared* (see Figure 2). Further research would be needed to explore which experts, and their ideas, were included or at the different stages of the foresight exercises.^4^ However, one can see the role of experts in the foresight exercises as WHO using a “Epistemic Communities Approach” to experts inclusion in policymaking (45, p. 458). Here the rationale for the use of experts is that there is considerable uncertainty and interdependence on the issue at hand. Experts are therefore used to define the interests of an actor (in this case WHO) rather than propose solutions, frame the problem and solutions, or determine the most effective solution as is the case with the “Evidence-based Policy Making Approach”.

## 5 Discussion

Beyond the content of the strong and weak narratives identified in and between these policy documents, the purpose of this research was to also consider their function. As Prior (38, p. 832) claims, this allows for us to explore “what the documents do rather than what they say they do.” This recognises that narratives are central to how technologies are legitimised, funded, governed, and adopted (29). In addition, from a policy perspective this acknowledges that policy is not a solution to pre-existing problems but rather is a result of various interpretations seeking to shape the problem and solution (46). This perspective is particularly pertinent here given that the foresight exercises were designed to explore and shape the future of AI in global health, and to encourage and facilitate the exchange of competing interpretations of this future. It is the points of struggle and tension that are the focus of this section, reflecting how and why WHO attempts to frame the discourse of the future use of AI in global health, and the organisation’s current and future role in this regard. It is to three central points of tension to which we now turn.

### 5.1 Straddling strong vs. weak narratives

A key finding from the foresight exercises was that strong narratives were rare, and when they were found this was only in the introductory sections. Rather, it was the weak AI narratives that were dominant throughout the documents. This stands in contrast to the broader trend in strong and weak narratives that Bory et al. (8, p. 7) note, namely the discourse on AI being “strong in framing and weak in detail.” There is considerable detail around the weak narratives within the documents, and the strong narratives only serve to frame the need for the exercises. The use of foresight by WHO is interesting, as it is an approach which can be seen as purposely prioritising engagement with weak AI narratives. This can be seen as WHO shaping the discourse away from strong, externally driven narratives, instead focusing on the more controllable weak AI narratives. WHO’s actions in this regard should be contextualised within the broader shifting power relations in global health, such as the increasing prominence of non-traditional tech companies in global health (47, 48). It highlights a tension between healthcare being instrumentalised to reinforce strong AI narratives about the potential role of AI in society, and how this narrows the areas where AI can play a future role in global health (49, 56).

It is argued here, that the use of weak narratives serve to counter WHO’s perceived waning epistemic authority, by focusing on an area where WHO have a unique advantage and mandate. This is the organisation’s agenda setting role for the adoption of emerging technologies in global health. As discussed previously, WHO’s approval or disapproval of certain technologies is impactful in terms of uptake of AI technologies by healthcare actors. Weak AI narratives thus are a powerful tool for shaping the discourse of AI in global health in a space where WHO’s authority is being challenged by new non-traditional actors who are increasingly setting the agenda on global health through the use of strong AI narratives. This can be seen as WHO drawing on what Bory et al. (8) refer to as the pragmatic and symbolic ramifications of weak narratives.

Further, as Bory et al. (8) note, the importance of the connection between strong and weak AI narratives should be considered. We can see the connection between WHO’s use of weak AI narratives as being adversarial to strong ones. Yet, strong narratives are also drawn on to justifying the materialisation of the weak AI narratives in the foresight exercises, which in turn is used to contest external strong AI narratives. As such, the weak and strong AI narratives can be seen as being deeply interconnected, in line with the claims of Bory et al.s’ (8).

### 5.2 Siloing AI and hedging their bets

AI is siloed from other potential areas of innovation in WHO’s foresight exercises. This stands in stark contrast to the increasingly common position of state actors that AI is the only means to address a plethora of healthcare challenges (1). The broader techno-solutionist discourse on AI is that the technology can solve all societal problems and do so better than humans. This discourse is especially prominent in healthcare (24), with the increasing scale and scope of AI in the sector being seen as inevitable and overwhelmingly beneficial (1). However, the framing of AI within the WHO foresight exercises contests this dominant narrative. Rather than being a technology that can be broadly applied across healthcare systems to solve a range of problems, in the foresight documents, AI is presented as only having minimal impact on global health in the long term and only doing so in very specific areas.

However, this siloing is complicated by the reality that AI technologies can be deployed across a range of areas of application (though this does not imply equal effectiveness across domains). As such, AI is sometimes framed as an “enabler” for other innovations in the WHO foresight documents, as well as various AI technologies being identified as distinct innovations in and of themselves. This enabler status allows AI to maintain its techno-solutionist allure while sidestepping critical engagement, a discursive move that risks reproducing AI as self-evident, singular and uncontroversial, as Suchman (34) warned against.

The challenge of navigating the dominant techno-solutionist discourse on AI is one with which WHO have been grappling in their broader policy agenda (5). The challenge for WHO here can be understood as similar to that described as arising from technological promises (50). WHO seeks to simultaneously drive and shape the narrative on how and where AI should be adopted in global health. Yet, at the same time they have extremely limited control over the direction of its development. Framing AI as either an isolated innovation or as an enabler can thus be used to strategically navigate the tensions resulting from technological promises of the impact of AI in global health.

Further to this, the umbrella term of AI is broken down into specific AI technologies in the documents. In this regard, WHO had a selective focus on machine learning, deep learning, computer vision, and robotics, while other AI technologies, such as expert systems were not included, or only feature very minimally, such as NLP. This largely aligns with the dominant discourse about which areas of AI are or will be the most impactful across society. However it also undermines mature and well-functioning AI technologies currently supporting the healthcare sector. As Rip and Voß (37) note, while umbrella terms are useful means to mediate between science and society, and gain access to resources, their stabilisation requires limiting what falls under the term. Here we can see how this leads to WHO having various blind spots on AI in their foresight exercises as well as organisationally.

The focus on machine learning (and its subset deep learning) aligns with and reinforces the dominant discourse that machine learning is the most promising field of AI. However, this is only the latest swing of the pendulum in a long history of the various fields within AI research being favoured or sidelined (51, 52). Expert systems and NLP, for example, have been through a series of hype cycles. Heathfield (53) discussed this in relation to medical expert systems. Thus, the focus on machine learning, deep learning, computer vision, and robotics also speaks to the need for WHO to frame the future of AI in global health as distinct from previous AI fields which were perceived as failing to deliver, as well as to stake a claim in the fields where AI is having, and likely will have, the largest impact.

Lastly, The use of weak AI narratives as a policy tool in the foresight exercises is in line with broader organisational trends by WHO in their efforts to shape the global governance of AI in global health, and their role within this. WHO have been active in shaping the emerging global governance around AI, being one of the few international organisations to have developed a range of soft law and sector-specific guidelines on AI^5^ and supported actors with the implementation of these (6). The foresight exercises can thus be seen as part of a larger policy discourse where WHO is trying to move away from strong AI narratives by utilising weak ones as an avenue to exert their authority in key strategic areas of AI and global health. Doing so also serves to position WHO as a global authority in legitimatising various AI applications in global health, thus reinforcing the need for the organisation in the public discourse.

### 5.3 The use of foresight and experts to legitimise WHO’s strategic priorities

The final area that warrants reflection is how WHO use the foresight method, and the role of experts within this process. As discussed in section *2.2 WHO and foresight*, the organisation notes that foresight allows them, and their member states, to better anticipate future trends, as well as critically reflect on their current operations. As such, they are not simply framing the future discourse on AI in global health, but also the current one. Recognising that discursive constructions of narratives in the documents are not ideologically neutral, the selection of experts, methodologies, and the framing mechanisms employed in the foresight process, which were controlled by WHO, should be considered. These choices reflect and reinforce the organisation’s broader ethos, especially its longstanding emphasis on social determinants of health over purely technological interventions. As such, the weak AI narratives that emerge tend to foreground public health infrastructure/spending and robust governance rather than technological capabilities alone. AI is thereby positioned not as a standalone solution, but as something deeply embedded in existing systems and dependent on broader socio-political and economic structures.

In addition, as mentioned previously it is in weak narratives that WHO can maintain its epistemic authority in global health. Experts are used here to not only materialise, but also to legitimise these weak AI narratives. This is not to say that the global health experts involved in these exercises were passive participants; rather that they act as intermediaries in the co-production of AI futures, but one that is controlled by WHO. Through their participation, they help to materialise and legitimise visions of AI that inform WHO’s current and future policy agenda. The foresight exercises therefore can be seen as a space where the future of AI in global health is not predicted but constructed and contested, with WHO using it, and the legitimacy of the experts, to shape the discourse around their strategic organisational priorities.

## 6 Conclusion

This study explored WHO’s foresight exercises using the analytical framework of strong and weak AI narratives (8). It revealed that strong narratives were rare and confined to the introductory framing of the foresight documents. The exercises themselves purposely prioritise weak narratives, emphasising practical implementation, technical limitations, and ethical concerns of AI in specific areas of global health. Interestingly, AI itself is siloed from other trends and areas of emerging technologies, though at times this distinction is blurred with various AI technologies being framed as distinct innovations in and of themselves, but also as an enabler for a range of other innovations. The role of experts in these foresight exercises was also reflected upon, with them being seen as providing authority and legitimacy to WHO’s current and future policy on AI through the materialisation and legitimatisation of weak AI narratives.

Regarding what these documents do in the world, it is argued that WHO strategically uses foresight not only to predict the future of AI in global health, but also to construct and legitimise certain AI futures, and the organisation’s role in this future. Through expert selection, methodological design, and narrative framing, WHO maintains its epistemic authority by steering global discourses toward specific, manageable applications of AI that aligns with their organisational priorities. Weak narratives are used to maintain this epistemic authority of WHO by grounding AI in an area where WHO have a unique mandate and legitimacy, i.e., in shaping the adoption and governance of new technologies in global health.

The foresight exercises provide the space for these weak narratives to come to the surface, with them being designed to identify and critically engage with specific AI technologies and their areas of deployment. Within these documents we can see WHO’s contestation of broader techno-solutionist narratives of AI in global health, and the actors that propagate theses. As such the foresight exercises can be seen as a space where the conflict to shape the future of AI in global health, and WHO’s role in this, is being played out.

## Data Availability

Publicly available datasets were analysed in this study. This data can be found here: WHO Publications https://www.who.int/publications.

## Footnotes

^1^For brevity the examples of instances of strong and weak narratives have been edited to include only minimal text. In the data extraction phase instances were coded within sentences or paragraphs where this provided essential context.

^2^Other Innovation Groups included *Diagnostic Technology*, *Health Products and Drug Delivery Technology*, *Tissue Engineering and Regenerative Medicine*, *Molecular Biology: Cell, Immune and Gene Therapy*, *Public Health: Environment, Climate Change, Epidemiology and Surveillance, Nutrition and Health*, *Dissemination and Implementation, and Materials and Biomaterials, Prosthetics*.

^3^Specifically, “medicine and health sciences, life sciences, social sciences, computer and information sciences, engineering and technology, and education” (40, p. 5).

^4^Research on the strong and weak AI narratives proposed and discussed by the experts, rather than just the outcomes of these discussions presented in the foresight documents, would be a fascinating line of enquiry. However, doing so was not possible within the scope of this paper.

^5^For example, see *Guidance on Large Multi-Modal Models* (54) and *The Role of Artificial Intelligence in Sexual and Reproductive Health and Rights* (55).
